# Renal artery pseudoaneurysm after blunt renal trauma: report on three cases and review of the literature

**DOI:** 10.1590/1516-3180.2013.1315488

**Published:** 2013-10-01

**Authors:** Kleiton Gabriel Ribeiro Yamaçake, Marcos Lucon, Antonio Marmo Lucon, José Luiz Borges Mesquita, Miguel Srougi

**Affiliations:** I Resident, Department of Urology, Hospital das Clínicas (HC), Faculdade de Medicina da Universidade de São Paulo (FMUSP), São Paulo, Brazil.; II MD. Attending Physician, Department of Urology, Hospital das Clínicas (HC), Faculdade de Medicina da Universidade de São Paulo (FMUSP), São Paulo, Brazil.; III MD, PhD. Assistant Professor, Department of Urology, Hospital das Cl ínicas (HC), Faculdade de Medicina da Universidade de São Paulo (FMUSP), São Paulo, Brazil.; IV MD, PhD. Professor and Head, Department of Urology, Hospital das Cl ínicas (HC), Faculdade de Medicina da Universidade de São Paulo (FMUSP), São Paulo, Brazil.

**Keywords:** Hematuria, Renal artery, Wounds, nonpenetrating, Aneurysm, Embolization, therapeutic, Hematuria, Artéria renal, Ferimentos não penetrantes, Aneurisma, Embolização terapêutica

## Abstract

**CONTEXT:**

Renal artery pseudoaneurysm is a rare complication after renal injury but should be suspected whenever there is recurrent hematuria after renal trauma.

**CASE REPORTS:**

We present three cases of pseudoaneurysm after blunt renal trauma and a review of the literature. All patients underwent renal angiography. Two cases were diagnosed during the initial hospital stay due to hematuria, or in the follow-up period during recovery. One patient was hemodynamically unstable. Two patients successfully underwent coil embolization in a single session. In the other case, selective embolization was attempted, but was unsuccessful because artery catheterization was impossible. Procedural and medical success and complications were retrospectively assessed from the patients' records. The clinical presentation, treatment options and clinical decisions are discussed.

**CONCLUSIONS:**

Renal artery pseudoaneurysm may develop acutely or even years after the initial injury. Signs and symptoms may have a wide spectrum of presentation. Selective angiographic embolization is an effective treatment that reduces the extent of parenchymal infarction.

## Abstract (pt)

**CONTEXTO:**

Pseudoaneurisma de artéria renal é uma complicação rara após lesão renal, mas deve ser suspeitada quando houver hematúria recorrente após trauma renal.

**RELATO DE CASOS:**

Três casos de pseudoaneurisma após trauma renal fechado são apresentados, além de revisão da literatura. Todos os pacientes foram submetidos a angiografia renal. Dois dos casos foram diagnosticados durante a estadia hospitalar inicial, por hematúria ou no período de acompanhamento durante a recuperação. Um paciente estava hemodinamicamente instável. Dois pacientes foram submetidos a embolização com coil com sucesso em uma única sessão. No outro caso, a embolização seletiva foi tentada sem sucesso, devido à impossibilidade de cateterização da artéria. O sucesso médico e do procedimento e as complicações foram retrospectivamente avaliados a partir dos registros dos pacientes. A apresentação clínica, opções de tratamento e decisões clínicas são discutidas.

**CONCLUSÕES:**

Pseudoaneurisma de artéria renal pode se desenvolver agudamente ou até mesmo anos após o trauma inicial. Sinais e sintomas podem ter um amplo espectro de apresentação. Embolização angiográfica seletiva é um tratamento efetivo, reduzindo a extensão de infarto parenquimatoso.

## INTRODUCTION

Pseudoaneurysm or false aneurysm is a confined accumulation of thrombus and blood associated with disruption of one or more layers of an artery wall. It differs from a true aneurysm in that the latter contains all three histological layers of the arterial wall, whereas pseudoaneurysm contains less than three and often none of these layers.^(^ ^1^ ^)^

Renal artery pseudoaneurysm occurs most frequently as a complication of certain renal interventional procedures such as kidney biopsy, percutaneous nephrostomy, open or endoscopic surgeries on the kidney, or as a consequence of penetrating trauma. Occurrences following blunt abdominal trauma are rare.^(^ ^1^ ^)^ A few other cases that have been reported described presentations with flank pain, pulsatile abdominal or flank masses, hypertension or hematuria, and ultimately resulted in lifethreatening hemorrhage followed by nephrectomy or death.^(^ ^2^ ^,^ ^3^ ^)^

We retrospectively reviewed three cases of pseudoaneurysm after blunt renal trauma and conducted a review of the literature. These three cases were managed in our institution between 2008 and 2011.

## CASE REPORTS

## Case 1

**Figure 1:**
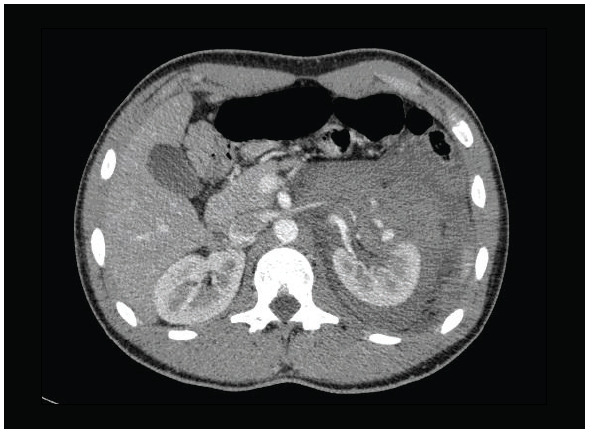
Computed tomography (CT): grade III left renal injury with large perirenal hematoma in Case 1.

A healthy 16-year-old male was admitted to our emergency department after a fall during a soccer game, with gross hematuria 30 minutes afterwards. He did not have any significant medical history. On physical examination, his blood pressure was normal and left upper quadrant abdominal tenderness was found. His hemoglobin was 11.6 g/dl and hematocrit was 28.8%. Grade 1 spleen rupture and grade 3 left renal injury with a large perirenal hematoma were revealed through abdominal computed tomography (Figure 1).

He was admitted to the intensive care unit for a careful check on vital signs, serial hematocrit monitoring and strict bed rest. Eight hours after admission to the intensive care unit, he presented tachycardia with normal arterial blood pressure. His hemoglobin level had dropped to 5.4 g/dl. He received 2000 ml of crystalloids and 3 U of packed red blood cells.

**Figure 2:**
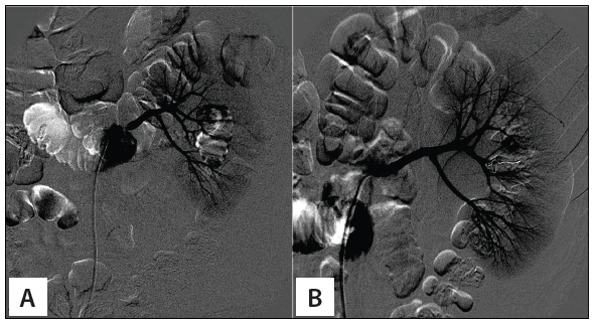
Angiographic confirmation of renal artery pseudoaneurysm (A) in Case 1 and result after embolization using microcoils (B).

The patient was transferred to the intervention radiology suite 16 hours after admission, where renal angiography showed a renal pseudoaneurysm that was then successfully treated by means of selective coil embolization (Figure 2). His hemoglobin level then stabilized.

A repeat computed tomography scan performed seven days after admission showed complete remission of the pseudoaneurysm and good kidney perfusion. The patient presented normal blood pressure and normal renal function, as assessed through a dimercaptosuccinic acid (DMSA) renal scan, during the six-month follow-up period.

## Case 2

A 25-year-old male patient was admitted to our emergency department after falling from a height of 15 meters. On admission, he presented a Glasgow coma score of 14, normal arterial blood pressure and a pulse rate of 100. Physical examination revealed hematuria and pain on neck palpation.

**Figure 3:**
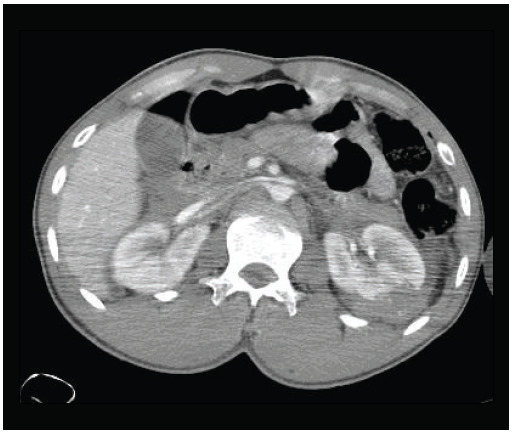
Computed tomography (CT) in Case 2: grade III left renal injury.

Infusion of four liters of crystalloid was prompted. Computed tomography revealed a cervical vertebra fracture and hip dislocation in addition to grade 3 left renal trauma with perirenal hematoma (Figure 3).

**Figure 4:**
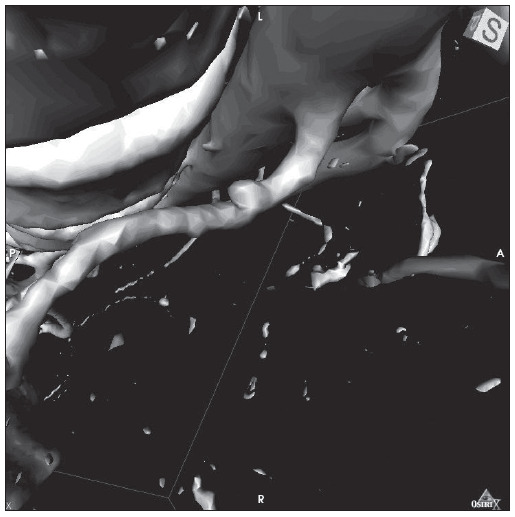
Three-dimensional reconstruction from computed tomography of Case 3: left renal artery pseudoaneurysm.

Conservative treatment for renal trauma was started, closed reduction was performed on the hip dislocation, a rigid neck collar was fitted and the hemodynamics were monitored. The patient evolved with hemodynamic stability and remission of the hematuria was observed four days later. A control computed tomography scan produced five days after the trauma showed that significant reduction of the perirenal hematoma had occurred and that there was a lesion suggestive of renal artery pseudoaneurysm (Figure 4).

The patient was then referred to the interventional radiology department and underwent angiography, which revealed a renal artery pseudoaneurysm that was successfully embolized using a coil. Information regarding follow-up was not available.

## Case 3

A 27-year-old patient was admitted to our emergency department after falling to the ground in a motorcycle crash. On admission, the patient presented hemodynamic stability and gross hematuria. Physical examination revealed left lower-back bruising. Subsequent tests showed fractures of the iliac wing and left twelfth rib. His hemoglobin level was 12 mg/dl, with hematocrit of 35%. A computed tomography scan showed a grade 2 left renal injury.

Conservative management with monitoring and hydration was started. Five days after the trauma, the patient was discharged home with mild hematuria that resolved spontaneously two days later.

Thirteen days after the trauma, a new episode of hematuria with urinary retention due to blood clots reduced the hemoglobin level to 10 g/dl. Fifteen days after the trauma, he underwent angiography, which showed a pseudoaneurysm. Selective embolization was attempted but was unsuccessful because artery catheterization was impossible. The patient was maintained under conservative treatment, without any decrease in hemoglobin level, but with hemodynamic stability.

He presented resolution of the hematuria sixteen days after the trauma, with recurrence thirty days after the trauma. A computed tomography scan was subsequently performed and showed hematoma regression. The scan ruled out the presence of any other significant lesions. Forty days after the trauma, the patient presented resolution of the hematuria and was discharged from the hospital. A computed tomography scan performed three months later was normal. 

## DISCUSSION

Renal artery pseudoaneurysm has most commonly been reported in relation to iatrogenic causes. Open and endoscopic surgery, renal percutaneous surgery and renal biopsy are the most frequent causes. Renal pseudoaneurysms have only rarely been described after blunt renal trauma.^(^ ^1^ ^,^ ^4^ ^)^ In this type of trauma, pseudoaneurysm formation is the result from the effect exerted by the deceleration forces on the artery.

The first case of renal artery pseudoaneurysm was described by Rouppe (1770, apud Rashid, 2007),5,6 who reported the demise of a sailor who fell on his right flank. The autopsy showed a large false aneurysm with rupture. Swana et al. reported on nine cases that had been published between 1770 and 1996.^(^ ^2^ ^)^ Few new cases have subsequently been reported.^(4,)(7,8)^

The clinical presentation includes hypertension, gross hematuria, lumbar pain and pulsating abdominal mass, but pseudoaneurysms may also be asymptomatic for a long time and develop spontaneous thrombosis.^(^ ^1^ ^)^ The average interval between injury and onset of the secondary renal hemorrhage is approximately 12 days (range: 2 to 36 days).^(^ ^9^ ^)^ Pseudoaneurysms are seen as thick-walled, well-defined accumulations adjacent to arteries, with an inner lumen that enhances after contrast administration, similarly to arteries. In addition, pseudoaneurysm walls may be covered by thrombi.^(^ ^10^ ^)^

Color Doppler ultrasonography may also be useful in diagnosing pseudoaneurysms. It has been shown to have very high sensitivity and specificity for detecting post-catheterization pseudoaneurysms,^(^ ^11^ ^)^ although its use in relation to visceral pseudoaneurysms is limited and depends on the lesion location and the operator's experience. Pseudoaneurysms present characteristic signs such as two-directional flow in the pseudoaneurysm neck and a "ying-yang" color pattern inside the lesion, thus revealing the direction of blood flow entering and leaving the pseudoaneurysm.^(^ ^12^ ^)^

Ultrasonography and computed tomography are particularly useful when renal vascular and parenchymal injuries are suspected. However, the ability to confirm the presence and anatomical location of renal artery pseudoaneurysms makes angiography the gold standard test of choice.^(^ ^13^ ^-^ ^16^ ^)^

We carried out a systematic analysis of indexed articles (Table 1). The search was performed using the terms listed in Table 1 in the Latin American and Caribbean Health Science Literature database (Literatura Latino-Americana e do Caribe em Ciências da Saúde, Lilacs), Excerpta Medica data-base (Embase), Medical Literature Analysis and Retrieval System Online (Medline) and Cochrane Library database, using Health Science Descriptors (Descritores em Ciências da Saúde, DeCS) and Medical Subject Headings (MeSH).

**Table 1:** Complete literature database search (on July 29, 2012) using terms corresponding to the main features of the patients reported

Database	Strategy	Related
Embase (via Ovid)	False aneurysm AND Renal artery AND Blunt trauma	Case reports: 11
		Descriptive series: 1
		Articles: 3
Medline (via Pubmed)	(("Aneurysm, False" [MeSH]) OR (Pseudoaneurysm) OR (Pseudoaneurysms)) AND ("Renal Artery"[Mesh]) AND (("Wounds, Nonpenetrating"[Mesh]) OR (Blunt Trauma) OR (Blunt Renal Trauma))	Case reports: 15
		Articles: 2
		Descriptive series: 1
Lilacs (via Bireme)	(Aneurysm False) OR ( Aneurisma falso) OR Pseudoaneurysm OR Pseudoaneurysms OR Pseudoaneurismas AND (Renal Artery) OR (Artéria Renal) OR (Arteria renal) AND (Wounds, Nonpenetrating) OR (Blunt Trauma) OR (Blunt Renal Trauma) OR (Ferida não penetrante) OR (Trauma Renal) OR (Heridas non penetrantes)	No results

Thirty-five cases (Table 2) of renal pseudoaneurysm after blunt renal trauma, indexed in PubMed, have been described.^(1-4),(6-8),(12),(17-34)^ The median age was 34.17 years (range: 11-79 years), and the majority were male (77.14%). The most common symptoms were abdominal/flank pain (11 out of 33 cases) and gross hematuria (23 out of 33 cases).

**Table 2:** Previously reported cases of renal artery pseudoaneurysms

Author	Age	GenderMale (M) Female (F)	Time between trauma and diagnosis	Diagnosis during hospital stay	Trauma location	Symptoms at diagnosis	Therapy	Result	Blood pressure	Creatinine	Follow-up
Chuang et al.^17^	35	M	7 days	Yes	Not reported	Gross hematuria and abdominal pain	Selective injection	Nephrectomy	Not reported	Not reported	Not reported
Lieberman et al.^18^	21	M	< 4 days	Yes	Left flank	Flank pain and gross hematuria	Gelatin sponge	Renal infarction	Not reported	Not reported	Not reported
Testart et al^.19^	54	M	36 months	No	Not reported	Abdominal pain	Bypass	Renal salvage	Not reported	Not reported	Not reported
Aburano et al.^20 ^	79	M	21 days	No	Not reported	Gross hematuria	Nephrectomy	Nephrectomy	Not reported	Not reported	Not reported
Steffens et al.^33 ^	15	M	12 days	Yes	Left flank	Gross hematuria	Embolization (2 x)	Renal salvage and 2nd embolization one year later	Normal	Normal	36 months
Swana et al.^2^	49	F	45 days	No	Not reported	Flank pain	Embolization	Renal salvage	Not reported	Not reported	Not reported
Farrel et al.^3^	27	M	9 days	Yes	Not reported	Hematuria	Not reported	Not reported	Not reported	Not reported	Not reported
Jebara et al.^21^	25	F	15 years	No	Not reported	Hypertension	Not reported	Not reported	Not reported	Not reported	Not reported
Jebara et al.^21^	17	F	8 years	No	Right flank	Abdominal pain	Not reported	Not reported	Not reported	Not reported	Not reported
Han et al.^7^	50	M	21 days	No	Right flank	Gross hematuria and hypertension	Not reported	Not reported	Not reported	Not reported	Not reported
Mizobata et al.^22^	40	F	10 days	Yes	Left flank	Not reported	Embolization	Renal salvage	Not reported	Not reported	Not reported
Mizobata et al.^22^	27	M	56 days	Yes	Back	Not reported	Embolization	Renal salvage	Not reported	Not reported	Not reported
Dinkel et al.^23^	28	F	8 days	Not reported	Not reported	Gross hematuria	Embolization	Renal salvage	Normal	Normal	2 years
Dinkel et al.^23^	34	F	3 years	Yes	Left flank	Gross hematuria	Embolization	Renal salvage	Normal	Normal	3 months
Dinkel et al.^23^	20	M	5 days	Not reported	Right flank	Gross hematuria	Embolization	Renal salvage	Normal	Normal	4 years
Dinkel et al.^23^	32	M	8 days	Not reported	Not reported	Gross hematuria	Embolization	Renal salvage	Normal	Normal	4 months
Miller et al.^4^	44	M	8 days	Yes	Not reported	Gross hematuria	Embolization	Renal salvage	Normal	Normal	3 months
Lee et al.^1^	32	M	18 days	No	Left flank	Gross hematuria	Embolization	Renal salvage	Normal	Normal	11 months
Lee et al.^1^	42	M	11 days	No	Left flank	Gross hematuria	Embolization	Renal salvage	Normal	Normal	9 months
Lee et al.^1^	38	M	45 days	No	Left flank	None (lesion discovered at follow-up by means of computed tomography)	Embolization	Renal salvage			
Lee et al.^1^	26	M	1 day	Yes	Abdomen	Shock	Laceration repair on post-injury day 1	Nephrectomy	Normal	Normal	6 months
Lee et al.^1^	24	M	1 day	Yes Diagnosis after surgery	Abdomen	Gross hematuria	Laceration repair on post-injury day 1 and embolization on post-injury day 2	Renal salvage	Normal	Normal	5 months
Halachmi et al.^24^	11	M	7 days	Yes	Left flank	Gross hematuria	Embolization	Renal salvage	Normal	Normal	1 month
Giannopoulos et al.^25^	25	M	7 days	Yes	Back	Gross hematuria and back pain	Embolization	Renal salvage	Not reported	Not reported	Not reported
Chatziioannou 2004^26^	23	M	1 day	Yes	Not reported	Hematuria	Embolization	Renal salvage	Normal	Normal	23 months
Saad et al.^12^	11	F	21 days	No	Right flank	Hematuria	Embolization	Renal salvage	Not reported	Not reported	Not reported
Lee et al.^8^	52	M	21 days	Yes	Not reported	Microscopic hematuria and flank pain			Not reported	Not reported	Not reported
Poulakis et al.^27 ^	24	M	60 days	No	Not reported	Microscopic hematuria and flank pain	Embolization	Renal salvage	Not reported	Normal	6 months
Guerra Requena et al.^28^	51	M	16 months	No	Not reported	Flank pain	Angiography: unsuccessful	Open surgery suture, renal salvage	Not reported	Not reported	Not reported
Pastorín 2007^29 ^	25	M	4 months	No	Abdomen	None	Embolization	Renal salvage	Normal	Normal	3 months
Rashid et al.^6^	49	F	49 days	No	Not reported	Gross hematuria			Not reported	Not reported	Not reported
Garg et al.^30^	30	M	9 years	No	Right flank	Gross hematuria	Embolization: unsuccessful	Renal salvage	Not reported	Not reported	Not reported
Lindekleiv et al.^31^	58	M	9 yeas	No	Right flank	Gross hematuria and flank pain	Embolization: unsuccessful	Nephrectomy	Not reported	Not reported	Not reported
Steinway et al.^32^	19	M	16 days	No	Abdomen	Gross hematuria	Embolization: unsuccessful	Nephrectomy	Not reported	Not reported	Not reported
Jackson et al.^34^	59	M	16 days	No	Abdomen	Gross hematuria and flank pain	Embolization	Renal salvage	Not reported	Not reported	6 months

Nonetheless, renal artery pseudoaneurysm may be asymptomatic. Lee et al. described the case of a 38-year-old male whose pseudoaneurysm was diagnosed 45 days after trauma during computed tomography follow-up.^(^ ^1^ ^)^ Pastorín et al. described the case of a 25-year-old male with an asymptomatic renal artery pseudoaneurysm that was observed four months after injury.^(^ ^29^ ^)^ One of the three patients in our series was diagnosed as presenting an asymptomatic pseudoaneurysm, during a follow-up examination four days after the trauma.

In the majority of the reports, the time that elapsed between the trauma and the diagnosis ranged from one day to 15 years (median of 28.4 months). In the literature, the longest time taken to make the diagnosis was reported by Jebara et al., in the case of a 25-year-old female who developed abdominal pain and hypertension 15 years after trauma.^(^ ^21^ ^)^

Most of the cases remained undiagnosed during the initial hospital stay (18 out of 31 cases). Two of our cases were diagnosed during the hospital stay due to hematuria or in the follow-up period during recovery.

Although clinically silent, small pseudoaneurysms may be managed conservatively. However, because of the risk of spontaneous rupture and mortality, many physicians recommend surgical management. In the past, surgical exploration or nephrectomy was used as the only treatment. However, recent advances in interventional radiological techniques have enabled superselective catheterization.^(^ ^1^ ^)^ Thus, nowadays, pseudoaneurysms can be treated with minimally invasive procedures such as embolization using coils, either alone or in combination with other materials like non-resorbable glues or onyx, which result in rapid hemostasis and more effective preservation of kidney function.^(^ ^35^ ^)^

The complication rate from the selective radiological embolization procedure itself is relatively low. Since 2001, 23 cases have undergone embolization with successful renal salvage in 20 cases (86.9%). Our cases were also successfully treated with embolization. Renal artery dissection has been described in up to 7.5% of the cases,^(^ ^36^ ^)^ whereas the incidence of coil migration is less than 3%.^(^ ^23^ ^)^

## CONCLUSIONS

Renal artery pseudoaneurysm is a rare complication following blunt renal injury. It may form acutely or may even be seen days, weeks or years after the initial injury. Although some cases may develop symptoms, others may remain asymptomatic even over the long term. Computed tomography and ultrasound scans can provide the diagnosis; confirmation and treatment are performed by angiography. The increasing use of conservative management for renal trauma has led to a higher rate of suspicion of renal artery pseudoaneurysm. This diagnosis should be considered whenever there is a recurrent bleeding after renal trauma.
